# The Training of Recruits for Nursing Orderlies

**Published:** 1917-12-22

**Authors:** Gilbert I. Strachan

**Affiliations:** 3rd Western General Hospital, Cardiff


### THE TRAINING OF RECRUITS FOR NURSING ORDERLIES.

By GILBERT I. STRACHAN, M.D., M.R.C.P., Captain R.A.M.C. (T.F.), 3rd Western General Hospital, Cardiff

Some few months ago the authorities of this hospital were informed by the Headquarters of the Western Command at Chester that periodical drafts of R.A.M.C. recruits were to be furnished to them to be trained and made efficient for hospital service abroad. There was no actual hard-and-fast period set in which to complete the training, but we could count fairly securely on having, at least, six weeks, and in all probability two months, in which to change the rawest recruit into a fully trained and equipped hospital orderly, and it will at once be appreciated that if such a metamorphosis were to be satisfactorily accomplished in the stated period or anything like it, no time was to be lost, and no energy spared in the matter.

It soon became clearly evident that if our efforts were to accomplish anything in the way of success haphazard methods must be utterly discarded, and a clear and rational line of procedure be followed. As the result of such considerations, and after consultations between the adjutant, the junior medical officers, and the heads of the nursing staff, the following scheme of training was agreed upon. The training of the men on the lines presently indicated is now proceeding, and it may be fairly stated that the results so far as can at present be judged are most encouraging, the men showing daily increasing understanding of their duties and alacrity in their performance.

The general scheme of training is divided into three parts:—(1) Stretcher and ambulance training; (2) theatre training; (3) ward training.

### Stretcher and Ambulance Training.

(1) For the purpose of this part of the training an ambulance is provided, and in the yards of the various sections of the hospital the recruits are assembled and instructed by the wardmaster as to the proper way in which to stand when proceeding to unload an ambulance; how, when the foot half of the stretcher is out two men have to be at hand to catch and lift clear the head end; how, when one stretcher has been so removed, another four men should at once be in place to attend to another stretcher, and so on.

They are drilled and drilled at this, and in a short time become very efficient indeed, and when a convoy of wounded arrive they can empty the vehicles in a quick and satisfactory manner, and with a minimum of fuss.

### Theatre Training.

(2) For theatre instruction the men are brought to the operating theatre at a time when it is not being used. A dummy is provided in the shape of one of themselves or an obliging convalescent patient, and instruction is administered in the various theatre technicalities—how to lift a helpless and prone patient from trolley to operating table; how to lift an anæsthetised patient after operation in the reverse direction; how properly to restrain a struggling man; how to conduct a case after operation back to his ward; these manœuvres are gone through time and again, and a considerable degree of celerity and slickness is soon attained. This rehearsing is put into performance, during actual operations, when the recruits are assembled and required to put into practice with actual patients these liftings and holdings which I have enumerated above.

This is found to work very satisfactorily indeed, and the recruits are soon very efficient in these exercises.

### Ward Training.

(3) But it is this third part which is the longest and hardest part of the training, for it has to go on side by side with the ordinary work of the wards.

The twelve instructions on this point contained in the official R.A.M.C. Instruction Book taken by us as the basis of procedure in the matter are as follow:—

1. Personal cleanliness, reasons why, washing of hands, keeping nails cut short.
2. Cleaning of wards, dusting, sweeping, cleaning baths, annexes, utensils, brasses, taps, disinfecting and cleaning of beds, mackintoshes, etc.
3. Ward kitchens, serving meals, etc., cleaning and washing-up of utensils.
4. Bedmaking, drawsheets, changing helpless patients. Washing helpless patients, lifting and moving helpless patients.
5. The correct reading of measure glasses and measures used.
6. Taking temperatures, pulse and respirations. Recording observations on charts.
7. Names of instruments in constant use in the wards. Syringes, enemas, and how to use them.
8. Lotions, varieties and strengths. Applications of wet and dry dressings, fomentations, compresses.
9. Methods of giving medicines, necessity of punctuality and exactness in administration.
10. Surgical dressings. The necessity of absolute cleanliness, sterilisation, disposal of soiled dressings.
11. Prevention of bedsores, care of bedsores, uses of air and water beds and cushions.
12. The nursing of head and spinal injuries and of patients suffering from paralysis, fractures. Nursing of enteric, disinfection of stools, urine, etc. Nursing of heart cases, rheumatism, nephritis, surgical cases, special medical cases.

Note.—Recruits are to be trained in medical and surgical wards.

A glance at this list will show that it is absolutely comprehensive and entirely covers any ground of knowledge which a nursing orderly can be expected to traverse.

In the morning from 7.30 a.m. until 10 a.m. paragraphs 1 to 3, as above, are got over, or at least as much of them as possible. In this period there are to be done these duties: clearing of breakfast dishes; sweeping and dusting of wards;

**THE TRAINING OF RECRUITS FOR NURSING ORDERLIES**—(*continued*).

attention to the requirements of patients (bed-pans, etc.); sweeping and dusting of corridors and the cleaning of brass; cleaning of bathrooms and the contained utensils; and in the sluice-rooms the cleaning of sinks, shelves, and brasses. From this it will be seen that the morning is well and busily occupied.

Between 10 A.M. and 12 noon demonstrations are given in the wards on the following amongst other subjects:

If a small ward be empty, so much the better; but if not, an empty bed in an occupied ward has to suffice. Bed-making; changing draw-sheets from under helpless patients, *e.g.* spinal cases; the proper cleansing and general attention of the skin in order to prevent bedsores; the accurate taking of temperatures; the proper application of the bed-pan and urinal; how properly to give an enema; the acute necessity for scrupulous cleanliness in all matters relating to surgical procedures; these points are especially emphasised and constantly reiterated.

A glance at the list given above will show that the area to be covered is wide, and that the period of six or eight weeks which I mentioned is pretty fully occupied in bringing the men to anything like a condition of efficiency.

The dinner-hour is from 12 noon until 1 P.M., and in the afternoon the men are engaged in ward work, helping the regular orderlies and simultaneously putting into practice the instruction which they receive.

Charge Sister's Efficiency.

These ward demonstrations are given mostly by the charge sisters of the various wards, but at intervals the junior medical officers give a demonstration and see how the instruction is progressing. These M.O.s are held responsible by the hospital authorities for the carrying out and for the efficiency of the instruction, in which, indeed, they take part as often as their other duties will allow. One cannot praise too highly the efficient manner in which the charge sisters perform their arduous and fagging duties in this respect.

Recruits in Training.

These recruits are not included in the list of men for ordinary duty, so that they can be assembled at any convenient time for any particular demonstration without in any way hampering the ordinary hospital work. Again, such time and energy as are spent by the staff on these demonstrations is such as can be snatched in the course of the ordinary hospital duties, which, necessarily, must first of all be attended to: no duties are excused for the purposes of these demonstrations.

The scheme such as I outline in skeleton above is now working very satisfactorily, and the recruits are obviously increasing in efficiency. I trust that these particulars may be of some interest to your readers. I furnish them with the permission and by the authority of Colonel Hepburn, V.D., C.M.G., the officer commanding this hospital.

